# Beyond high cost: Pharmaceutic low-resource setting barriers to antibacterial access

**DOI:** 10.1371/journal.pgph.0004280

**Published:** 2025-03-18

**Authors:** Marvellous O. Adeoye, Tochukwu C. Agboeze, Joseph A. Adegoke, Iruka N. Okeke

**Affiliations:** 1 Department of Pharmaceutical Microbiology, Faculty of Pharmacy, University of Ibadan, Ibadan, Nigeria; 2 Department of Medical Microbiology and Parasitology, College of Medicine, University of Ibadan, Ibadan, Nigeria; PLOS: Public Library of Science, UNITED STATES OF AMERICA

Antimicrobial resistance (AMR) is a large and growing threat, killing an estimated 1.14 million people in 2021 [1]. The situation is most dire on the African continent, where the AMR burden is exacerbated by high infectious disease burdens and low access to effective medicines. While much attention has been given to the diminished antibiotic development pipeline, and the high cost of new medicines [2], a less-discussed yet critical issue worsening access for African patients arises from the chemistry and formulation of antibacterials. Orally active, broad-spectrum ‘Access’ [3] antibacterials that can be stored for extended periods at room temperature have seen the greatest use in hot, humid African settings with weak supply chains. In the most challenging settings, optimal ‘room temperature’ storage conditions (20–25**°**C) typically require air-conditioning that is absent or intermittent [4,5]. Additionally, durations between shipments can be long. Even under these conditions, sufficient activity for efficacy is retained for most of the shelf-lives of many orally active beta-lactams, tetracyclines, antifolates, quinolones, and chloramphenicol licensed before 1990. Pediatric multidose reconstitutable powders pose special challenges when refrigeration is unavailable, but health post-clinic-based bottle-sharing can ensure that new reconstitutions are made close to the time of use. Orally active antimicrobials do not require delivery equipment but can be used by community health workers, thereby overcoming to some degree the limitation that antimicrobial options are fewer for those who cannot or will not be admitted to the hospital [6].

Another important consideration for access is activity spectrum. The deeper diagnostic information essential for the rational use of narrow-spectrum antimicrobials can make them inaccessible in the absence of laboratory infrastructure or point-of-care testing [7]. Thus, until compromised by resistance, orally-active, broad-spectrum antimicrobials underpinned successful antibacterial chemotherapy for most patients seeking care in remote African and similar settings for two-thirds of a century.

## The antibacterial development pipeline is biased away from accessible options

The slow pace of antimicrobial discovery and development is a large contributor to the present resistance crisis. However, attempts to replace the antibacterials of a generation ago with less thermally stable alternatives and drugs that require more clinical expertise for use, exacerbate inaccessibility in remote African settings. We observe that in contrast to the mid-to-late 20^th^ century, a significant proportion of antimicrobials in use today need to be stored refrigerated in the long term, or at reconstitution. Beta-lactams, the most frequently used antibacterials worldwide, have excellent safety profiles and are popular choices for neonatal sepsis, typhoid fever, and other hospital and community-acquired infections common in Africa [8]. Only five beta- lactams have been licensed since 2018 and three of them, cefidericol (2019), sulbactam-durlobactam (2023), and ceftobiprole (2024) must be refrigerated.

**Fig 1:**
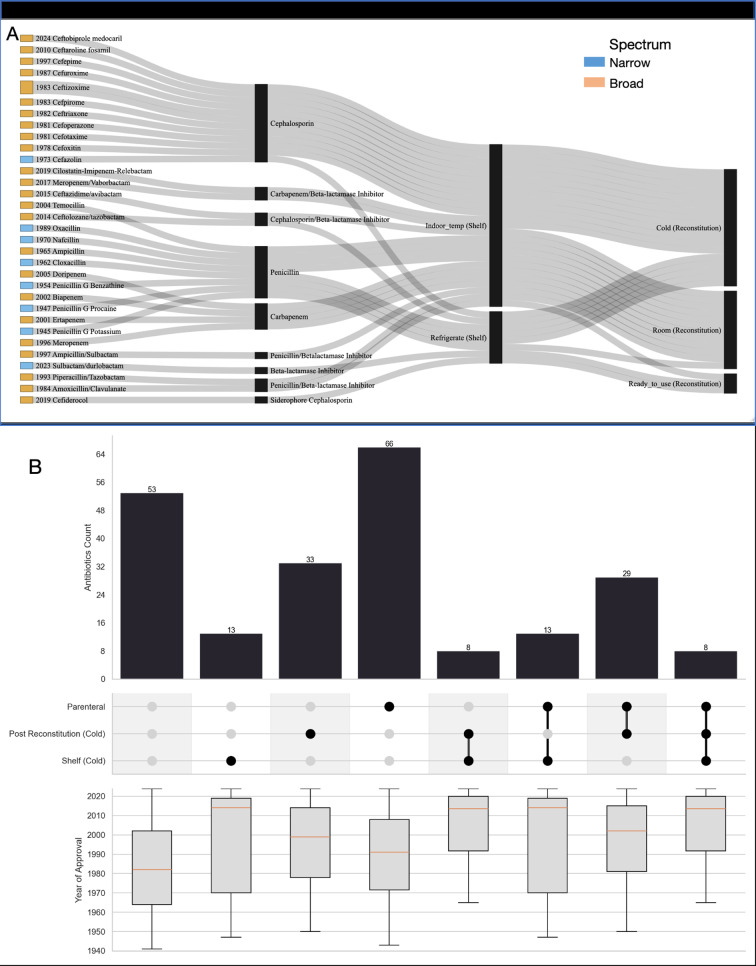
A. Sankey plot listing Beta lactam antibacterials in current use showing columns (left to right) for the year of introduction and generic name of the antimicrobial; the beta lactam subclass; the formulated medicine’s shelf storage, and the storage conditions required after reconstitution at use. B. UpSet plot showing the number of in-use antibacterials that are parenteral, require cold storage after reconstitution or on the shelf, and all combinations of those. Below the UpSet plot is a box plot illustrating the distribution of approval years for antibacterials in each category. The interquartile range (IQR) is represented by the shaded boxes, while the whiskers indicate the absolute range of approval years. The median approval year is marked by an orange horizontal line within each box.

Cephalosporins, while less prone to hydrolysis than penicillin beta-lactams [9], still require cold storage to ensure stability, particularly after reconstitution (Fig 1a). Carbapenems are shelf-stable at room temperature but require refrigeration or prompt use once reconstituted. Peptide antibiotics (except telavancin), fluoroquinolones, and oxazolidinones generally remain stable at room temperature. However, these examples are exceptions. When we plot the median year of approval, as in Fig 1b, a prominent trend toward refrigeration requirements for both shelf and post-reconstitution storage can be seen.

Cold storage is often presented as a barrier to vaccine access but is overlooked in antimicrobial stewardship discourse, which largely focuses on prescription rates. Patients and communities dependent on rural health centers and small pharmacies without consistent refrigeration, risk reduced efficacy and the potential exacerbation of antimicrobial resistance for thermolabile antibacterials.

Remote health facilities may also lack skilled caregivers, and the scarcity of health workers in Africa is difficult to rectify with current migration trends. Fig 1b shows that the median year of approval for antimicrobials requiring *both* cold shelf storage and parenteral administration is 2014. For antibacterials with neither of these requirements, it is 1982. In least-resourced settings where electric power supply is absent or intermittent [4] and doctors and nurses are in short supply, the need for cold chain storage, parenteral administration, or both, poses major barriers to equitable access. Health facilities lacking the resources to use newer antibacterials are disproportionately located in African countries, which bear the highest infectious disease burden and have the least capacity for patient referral [10]. Between the 1980s and early 2000s, 40 (83%) approved antimicrobials were broad spectrum agents but in contrast, 9 (35%) non-tuberculosis antibacterials approved from 2010 have narrow spectra (S1 Fig). Narrow-spectrum agents were more common early in the 20^th^ century and the recent return to developing them offers significant advantages, since they do not select for resistance broadly. However, unless narrow-spectrum antibacterial development is paired with diagnostic innovation, they will not be accessible. Since 2017, only 13 antibiotics have been approved [11], with lefamulin (2019) being the sole new chemical class, and also narrow-spectrum. Of the four antibiotics approved in the 2020s, two—contezolid (2021) and pivmecillinam (2024)—are narrow-spectrum oral agents, requiring laboratory support for judicious use (S1 Fig).

## Moving forward: A call for integrated development of accessible antimicrobials

It is an unfortunate reality that electric power and diagnostic access requirements remain unaddressed in African health systems [4] where resistance to earlier ‘Access’ antibiotics is commonplace. Many patients will not access newer ‘Watch’ and ‘Reserve’ drugs, even if they could afford them. Innovation in pharmaceutics must accompany active-principle drug development to ensure that newer antibacterials can be used when drug resistance mandates them. Expanding antibacterial access could also be enabled by investments in solar- or wind- powered refrigeration or modernized versions of historically used cooling systems like clay pots, which can maintain lower temperatures in the tropics without active refrigeration [12]. Stability studies in and for Africa, similar to those executed for COVID-19 vaccines [10], could reveal that some newer antibiotics withstand less-than-ideal storage conditions, potentially improving access. Integrating these storage solutions into the supply chain could significantly improve antibiotic availability and effectiveness in Africa and other LMICs, enhancing global AMR management. Climatic data collection across Africa could provide critical insights for innovators, manufacturers, regulatory agencies, and health systems, helping to establish appropriate storage guidelines tailored to the unique challenges in the tropics [13].

Multifaceted solutions to the AMR crisis must include the development of new antibiotics as well as infrastructure to support their safe and effective use. Some barriers we lay out here have been articulated for select antimicrobials [14]. However, the trends we present suggest that if they are not holistically addressed, discovery will not overcome antibacterial access barriers in multiple resource-constrained settings, and therefore the computed burdens from AMR will rise. Laxminarayan *et al*. (2024) observed that rising costs in antimicrobial development and slow progress in affordable diagnostics are major barriers to access [2]. Here, we lay out other hurdles to access that must feature in future Target Product Profiles, and be addressed by developers to ensure next-generation antimicrobials reach patients across Africa and other low-income tropical settings. As for vaccines, the push for investment in antibiotic development and healthcare infrastructure must include robust cold chain and storage systems, or prioritize medicines that can be delivered without them [10]. The need to envision formulations other than parenteral is similarly pressing. Dosage forms like suppositories, as used for some antimalarials, skin patches, or intranasal options should be explored wherever oral administration is infeasible. Patient and system needs must be prioritized if antimicrobial resistance is to be equitably addressed.

## Supporting information

S1 Fig Number of antibacterials approved in each decade from the 1940s to the 2020s that are classed as anti-tuberculosis drugs, other narrow spectrum antibacterials or broad spectrum antibacterials. (TIFF)
