# Supplementary figures and images for: Beyond high cost: Pharmaceutic low-resource setting barriers to antibacterial access
Source: PLOS Glob Public Health. 2025 Mar 18;5(3):e0004280. doi: 10.1371/journal.pgph.0004280 (PMC11918388; doi:10.1371/journal.pgph.0004280)

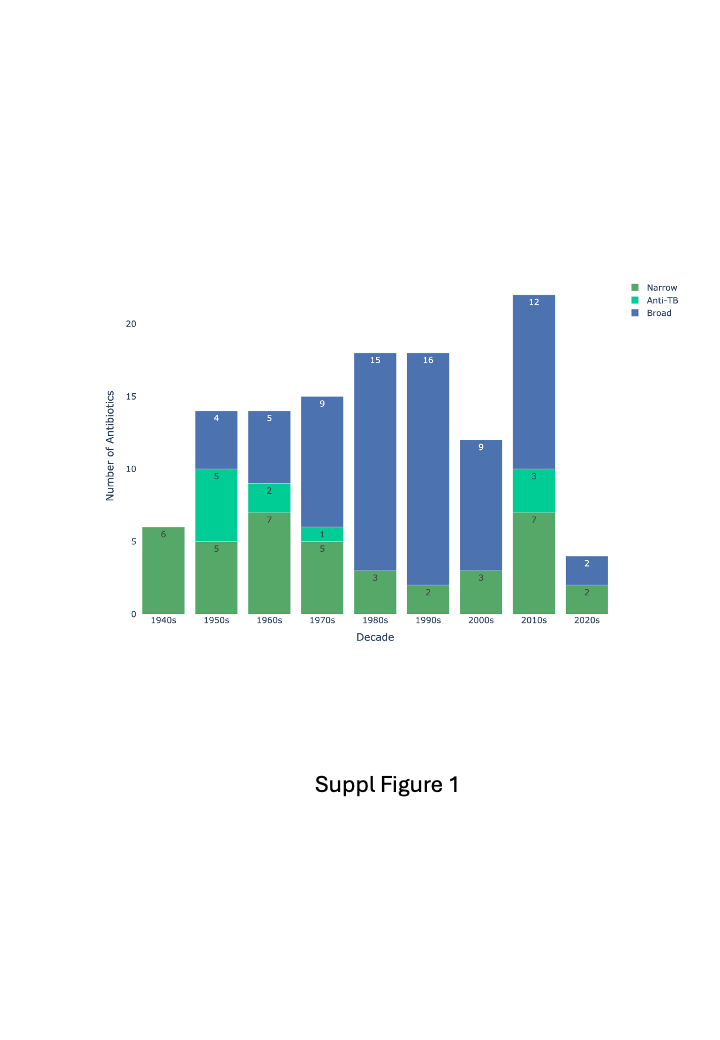

Supplement: S1 Fig — (TIFF) [file pgph.0004280.s001.tiff]
